# Anthocyanin Fluorescence for Monitoring Oxidation of the Chokeberry Fruit During Drying and Storage of Fruit Lyophilizates

**DOI:** 10.1002/fsn3.72223

**Published:** 2026-09-03

**Authors:** Małgorzata Rak, Grzegorz Bartosz, Kacper Kut, Ireneusz Kapusta, Maria Goc, Izabela Sadowska‐Bartosz

**Affiliations:** ^1^ Laboratory of Analytical Biochemistry Institute of Food Technology and Nutrition, Faculty of Technology and Life Sciences, University of Rzeszow Rzeszow Poland; ^2^ Doctoral School University of Rzeszow Rzeszow Poland; ^3^ Department of Food Technology and Human Nutrition, Institute of Food Technology and Nutrition, Faculty of Technology and Life Sciences University of Rzeszow Rzeszow Poland

**Keywords:** anthocyanins, *Aronia melanocarpa*, chokeberry, drying, fluorescence, lyophilizate

## Abstract

The appearance of the “oxidation band” in the fluorescence spectrum of anthocyanins is an indicator of oxidative deterioration of anthocyanins and can be a useful preliminary index of food freshness. Chokeberry ( *Aronia melanocarpa* ) fruit, rich in anthocyanins, can be subject to drying or lyophilization for domestic or industrial applications. The study was aimed at determining whether the measurement of fluorescence of dried chokeberry extracts can provide information on the extent of anthocyanin oxidation during prolonged drying at 60°C, and if fluorescence of fruit extracts can monitor anthocyanin oxidation during storage of the lyophilizates at various temperatures under air exposure. The increase in the fluorescence intensity of the extracts of chokeberry fruits or their lyophilizates at the “oxidation band” was a regular function of the time of fruit drying and the time of lyophilizate storage at 22°C and 37°C, confirming the usefulness of this parameter for estimation of oxidative changes in dried fruits and their lyophilizates. However, fluorescence of whole fruits, pulverized fruits and pulverized lyophilizate did not show a regular increase as a function of the time of fruit drying and lyophilizate storage, apparently due mainly to the effects of light scattering. The results point to the necessity of controlling the drying process of chokeberry fruits and storing their lyophilizates at ambient or higher temperatures with air access to avoid anthocyanin oxidation.

## Graphical abstract

An increase in the fluorescence intensity of anthocyanins in the extracts of chokeberry fruits or their lyophilizates at the “oxidation band” (520–530 nm for the excitation wavelength of 460 nm) was a regular function of the time of fruit drying at 60°C and of the time of lyophilizate storage at 22°C and 37°C with air access, confirming the usefulness of this parameter for estimation of oxidative changes in dried fruits and their lyophilizates.

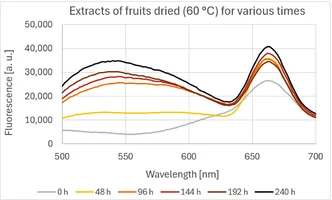

## 1 Introduction

Black chokeberry ( *Aronia melanocarpa* (Michx.) Elliott) is a perennial deciduous shrub of the Rosaceae family (Maloideae subfamily), native to eastern North America (Kaloudi et al. 2022; Duman et al. 2025). At the beginning of the 20th century, it was introduced to some European countries, including Russia, Poland, Germany, Norway, and Finland, and then, in the second half of the same century, it also became widespread in East Asia, including Japan (Piras et al. 2024).

*A. melanocarpa* fruit is spherical; its diameter ranges from 6.1 to 17.8 mm, depending on the variety and its weight ranges from 0.5 to 2 g (Ochmian et al. 2012). On ripening, the black chokeberry fruit takes on a deep, dark purple color, due to the high anthocyanin content (Kaloudi et al. 2022). The berry has a bitter and tart flavor (Wilkes et al. 2014).

Black chokeberry is considered one of the richest plant sources of bioactive compounds, demonstrating high nutritional and biological value. The dominant component of the total phenolic compounds in *A. melanocarpa* is proanthocyanidins, which occur in a highly polymerized form, composed primarily of polymers formed by varying numbers of catechin units (including epicatechin) linked by C–C bonds. Under the influence of factors such as increased temperature, acidic environment, or alcohol, these proanthocyanidins are converted to cyanidin, which leads to a significant increase in antioxidant activity (Shi et al. 2024).

The second largest group of phenolic compounds present in black chokeberry is anthocyanins. They constitute 25%–50% of the total phenolic content, and their concentration on a dry weight basis ranges from 0.6% to 2% (Jurendić and Ščetar 2021; Zhang et al. 2021). The main anthocyanins found in *A. melanocarpa* fruit are cyanidin‐3‐*O*‐galactoside, cyanidin‐3‐*O*‐glucoside, cyanidin‐3‐*O*‐arabinoside, and cyanidin‐3‐*O*‐xyloside, with the dominant compound, cyanidin‐3‐*O*‐galactoside, accounting for almost 70% of the total anthocyanin content (Veberic et al. 2015; Olechno et al. 2022). Moreover, pelargonidin 3‐*O*‐galactoside and pelargonidin arabinoside were also detected in trace amounts (Oszmiański and Wojdyło 2005). The distribution of phenolic compounds in chokeberry berries is heterogeneous; the vast majority of anthocyanins, as much as 73%, are found in the peel, while the pulp contains most of the phenolic acids (78%) (Kaloudi et al. 2022). The rich chemical composition of black chokeberry fruit is not limited to phenolic compounds but also includes a wide spectrum of other nutrients such as vitamins C, B_2_, B_3_, B_9_, and K, tocopherols, carotenoids, as well as minerals such as calcium, phosphorus, magnesium, potassium, iron, copper, manganese, zinc, selenium, and iodine (Pavlović et al. 2015; Buda et al. 2020; Olechno et al. 2022). *A. melanocarpa* fruit belongs to the fruits with the strongest antioxidant activity (Kim et al. 2021).

The medicinal properties of the black chokeberry were appreciated already in ancient times by the indigenous people of North America, who used both the berries and the bark to prepare infusions for colds and as astringents (Gurčík et al. 2023; Xu et al. 2024). Due to increased nutritional awareness in recent years, *A. melanocarpa* berries have become increasingly popular among consumers (Nour 2022; Thorakkattu et al. 2025).

The edible parts of *A. melanocarpa* , mainly the fruit, have found widespread use in the food industry. Despite the growing popularity of chokeberries in recent years, the berries are rarely consumed in their natural, fresh, and unprocessed form due to their taste (Borowska and Brzóska 2016; Nour 2022). Considering the seasonal nature of harvesting and the short shelf life of fresh black chokeberries, processing is essential to ensure the availability of this valuable raw material and to enable the utilization of its bioactive potential year‐round (Sidor et al. 2021). The intense, dark purple color of the chokeberry fruit makes it suitable both as a source of natural food coloring and as a raw material with a high phytonutrient content used in the production of functional foods, the demand for which is growing due to its broad spectrum of beneficial effects beyond basic nutritional values (Lee et al. 2005; Šic Žlabur et al. 2017). Additionally, the *Aronia melanocarpa* fruit demonstrates technological potential as a natural preservative. The black chokeberry extract was found to inhibit the growth of many common pathogens, including *Staphylococcus aureus* , *Klebsiella pneumoniae* , and *Escherichia coli* (Paunović et al. 2022). The use of chokeberry extracts in meat, fish, and seafood products is effective in reducing lipid oxidation, maintaining freshness, and extending shelf life (Xu et al. 2024).

Taking into account the multiple applications of the chokeberry fruit, the estimation of the quality of its products is of considerable importance. Anthocyanins present in the chokeberry fruit provide color and protect against reactive oxygen species and excessive light and UV radiation to critical plant organs (Santos‐Buelga et al. 2014; Alappat and Alappat 2020; Zhao, Blum, et al. 2022a). They are sensitive to oxidation and degradation and thus relatively labile. Their color changes with pH, which makes them ideal potential candidates for internal markers of food freshness and quality. There are many examples of applications of anthocyanins to monitor food freshness (Wu et al. 2021; Zhao, Liu, et al. 2022b; Wang and Liu 2024). For example, a colorimetric indicator film based on *Aronia melanocarpa* has been developed, which allows visual monitoring of food spoilage through color change of anthocyanins, while simultaneously employing the antioxidant activity of these compounds to limit oxidation processes (Aydogdu Emir et al. 2023).

It seemed thus reasonable to expect that the oxidative degradation of anthocyanins may be a sensitive index of the freshness of the chokeberry fruit and food products based on this fruit. The degradation of anthocyanins can be monitored by measuring the decrease in their content, by estimating changes in the color of anthocyanin‐rich products, or by measurements of the formation of terminal scission products such as gallic and protocatechuic acids (Loypimai et al. 2016; Sinela et al. 2017; Chen et al. 2023). However, facile measurements of color changes may not bring unequivocal information on anthocyanin degradation since they may be influenced by changes in pH and in the association of anthocyanins with other compounds and metal ions (Chen et al. 2023), while studies of the appearance of degradation products require sample processing and advanced analytical methods such as HPLC (Sinela et al. 2017; Chen et al. 2023). Thus, a simple method allowing for a rapid and specific monitoring of anthocyanin oxidation could be useful for the food industry. We observed that oxidation of anthocyanins and anthocyanidins is accompanied by the appearance of a new fluorescence band centered at about 520 nm for the excitation wavelength of 460 nm (Bartosz et al. 2020). This fluorescence was proven to be useful in monitoring the extent of oxidation in such food products as juices, oils, and antioxidant‐rich fruit subjected to thermal treatment (Bartosz et al. 2020; Rak et al. 2025). The present study aimed to examine whether this fluorescence band can be useful in the estimation of anthocyanin oxidation during the drying of the chokeberry fruit and storage of lyophilized fruit.

While simple comparison of fluorescence intensity is useful in studies of changes in the oxidation state of a defined sample subject to storage or heating, estimation of the state of an unknown sample is more difficult since the initial fluorescence of a fresh sample is unknown. In such cases, a ratiometric parameter F_1_/F_2_ expressing the ratio of the “oxidation band” fluorescence to the native anthocyanin fluorescence would be more useful. Additionally, this parameter may be more sensitive as often an increase in the “oxidation band” fluorescence is accompanied by a decrease in the native fluorescence of anthocyanins (Bartosz et al. 2020; Rak et al. 2025). This parameter was also used to analyze the results whenever possible.

## 2 Materials and Methods

### 2.1 Materials

2,2′‐Azino‐bis(3‐ethylbenzothiazoline‐6‐sulfonic acid) (ABTS; CAS no. 504‐14‐6; cat. no. 10102946001; purity ≥ 99%) was provided by Roche (Warsaw, Poland). Iron(III) chloride (FeCl_3_; CAS no. 7705‐08‐0; cat. no. 451649; purity ≥ 99.99%) was obtained from MedChemExpress (Monmouth Junction, NJ, USA). 3,4,5‐Trihydroxybenzoic acid monohydrate (CAS no. 5995‐86‐8; cat. no. 398225), sodium carbonate (CAS no. 497‐19‐8; cat. no. 22353), hydrochloric acid (CAS no. 7647‐01‐0; cat. no. 1.13386), 2,4,6‐tri‐2‐pyridinyl‐1,3,5‐triazine (TPTZ; CAS no. 3682‐35‐7; cat. no. T1253), dipotassium sulfonatooxy sulfate (CAS no. 7727‐21‐1, cat. no. 216224) and the Folin–Ciocalteu phenol reagent (cat. no. 4764) were from Merck (Poznań, Poland). Glacial ethanoic acid (CAS no. 64‐19‐7; cat. no. JT9522‐2), sodium ethanoate anhydrous (CAS no. 127‐09‐3; cat. no. BN60/6191; purity ≥ 99%), and ethanol (CAS no. 64‐17‐5, cat. no. 396420113), 96% were provided by Avantor Performance Materials (Gliwice, Poland).

Black chokeberry ( *A. melanocarpa* (Michx.) Elliott) fruits grown in Jawornik, Niebylec commune, Subcarpathian Voivodeship, south‐east Poland, were washed and dried with a paper towel. The stalks were removed, and the fruits were heated in a mushroom/fruit drier (ESPERANZA Chanterelles EKD002, EDC Poterek, Ożarów Mazowiecki, Poland) at a temperature of 60°C ± 2°C, removing portions for analysis after 48, 96, 144, 192, and 240 h. Portions of the samples of fruit were pulverized in a grinder. Other portions were homogenized in ethanol (amounts corresponding to 1 g of initial wet mass/5 mL); the homogenate was centrifuged, and the supernatant was used for further analyses.

A portion of the fruit was lyophilized in a Harvest Right lyophilizer (Wuxi Marley Technology, Weihai City, Shandong Province, China) using conditions recommended by the manufacturer (initial freeze, 249.8 K; drying temperature, 324.8 K; drying time, 16 h). The lyophilizate was stored for up to 6 weeks at a cold room temperature (4°C ± 1°C), ambient temperature (21°C ± 1°C), and 37.0°C ± 0.5°C under the atmosphere of air for up to 6 weeks. After each week of storage, aliquots of the lyophilizate (70 mg) were dissolved in 5 mL of distilled water (15 min on a shaker), centrifuged, and the supernatant was used for analysis.

Distilled water was purified using a Milli‐Q system (Millipore, Bedford, MA, USA). Transparent flat‐bottom 96‐well plates (cat. no. 655101) and black flat‐bottom 96‐well plates (cat. no. 655209) (Greiner, Kremsmünster, Austria) were used for the assays.

**FIGURE 1:**
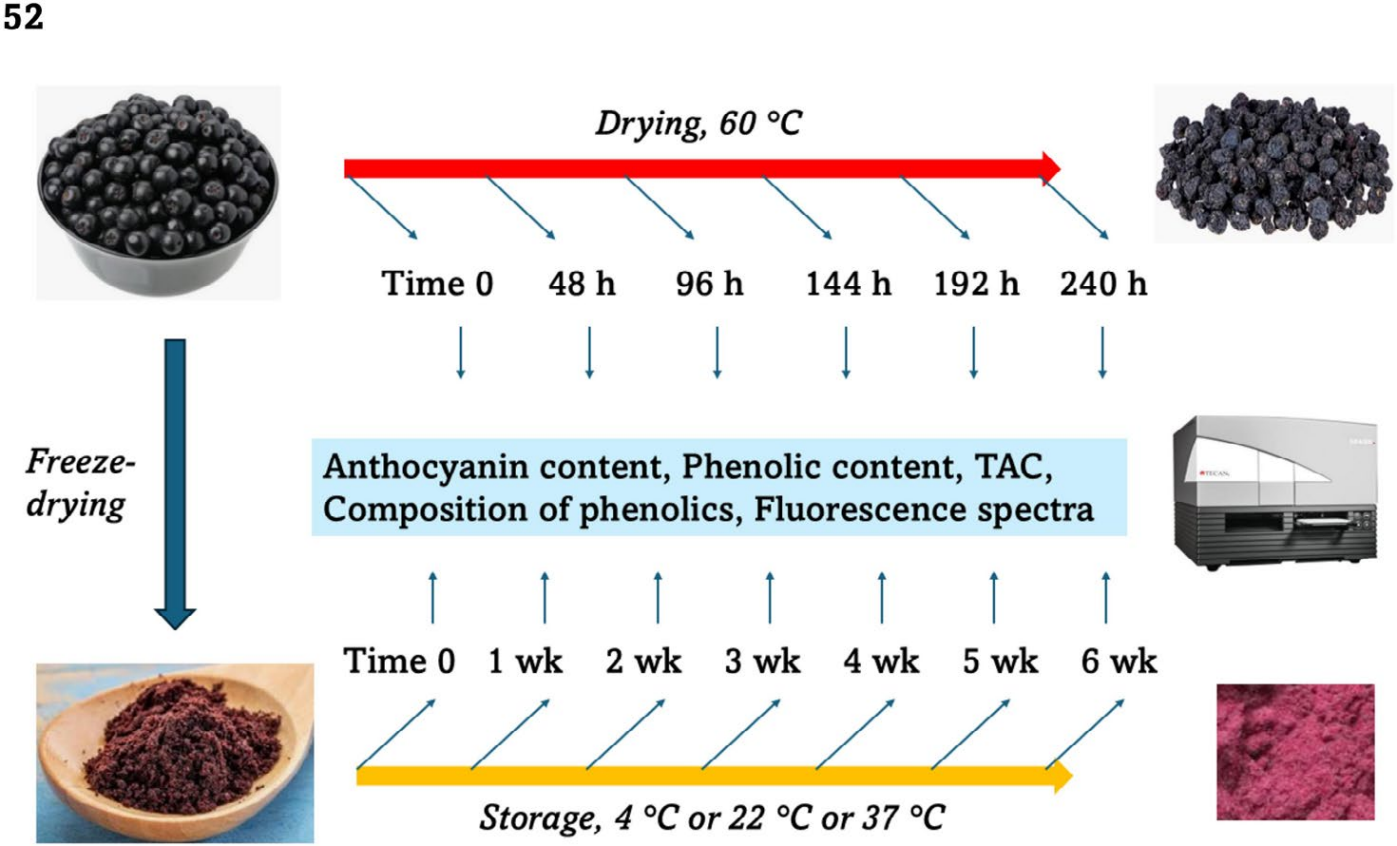
Scheme of the experiment.

A scheme of the experimental procedure is shown in Figure 1.

### 2.2 Estimation of the Phenolic Content

The phenolic concentration in the extracts was estimated using the Folin–Ciocalteu reagent (Fu et al. 2011). Briefly, 25‐μL aliquots of the extract (diluted 10 times with water) were added to 125 μL of 1 M Folin–Ciocalteu reagent in wells of a 96‐well plate. After 4 min, 100 μL of saturated disodium carbonate solution (about 75 g/L) was added. The absorbance of the mixture was measured at 750 nm after incubation for 1 h at ambient temperature. The absorbance reading was compared with the standard curve prepared using 3,4,5‐trihydroxybenzoic acid and expressed in 3,4,5‐trihydroxybenzoic (gallic) acid equivalents (GAE).

### 2.3 Estimation of the Anthocyanin Content

Anthocyanin content was estimated according to a slightly modified procedure of Lee et al. (2020). Briefly, 125 μL aliquots of the extract were added to 875 μL of 0.1 M acetate buffer, pH 4.5 or 1.5 M HCl, and the absorbance of both samples was measured at the absorption maximum (at about 520 nm) and 700 nm.

The anthocyanin concentration *c* was calculated as follows:

$$c\;\left[\text{μmol}/L\right]=A\times \text{dilution}\times 10^{3})/\left(\varepsilon \times l\right),$$

where

$$A={\left(A_{\max}-A_{700\;\mathrm{nm}}\right)}_{\mathrm{pH}\;1}-{\left(A_{\max}-A_{700\;\mathrm{nm}}\right)}_{\mathrm{pH}\;4.5},$$

with *A*_max_, maximal absorbance; *ε*, millimolar absorption coefficient for cyanidin‐3‐glucoside (*ε* = 26.9 mM^−1^ cm^−1^); and *l*, length of the optical path (cm). When 200‐μL samples were measured in a well of the 96‐plate used, the optical path was 0.577 cm.

### 2.4 Measurement of Total Antioxidant Capacity (TAC)

#### 2.4.1 The Ferric Reducing Antioxidant Power (FRAP) Assay

The method proposed by Benzie and Strain (1996) was slightly modified. Briefly, increasing volumes of solutions of the studied compounds were added to wells of a 96‐well microplate prefilled with 200 μL of the working solution composed of 0.3 M ethanoate buffer, pH 3.6 (10 volumes), 10 mM TPTZ in 40 mM HCl (1 volume), and 20 mM FeCl_3_ (1 volume), prepared immediately before use. After a 30‐min incubation at ambient temperature, the absorbance of the Fe^2+^‐TPTZ complex was read at 593 nm.

#### 2.4.2 ABTS ^•^ Decolorization Assay

A modification (Kut et al. 2022) of the ABTS^•^ decolorization assay (Re et al. 1999) was employed. Briefly, various volumes of an extract were introduced to wells of a 96‐well microplate, each prefilled with 200 μL of ABTS^•^ solution. The stock ABTS^•^ solution was prepared by overnight oxidation of 7 mM ABTS with 2.45 mM dipotassium sulfonatooxy sulfate (final concentrations). This stock solution was diluted with phosphate‐buffered saline (PBS) so that 200 μL of the sample had an initial absorbance of 1.0 at 734 nm in a well of a 96‐well microplate. The drop in absorbance was measured after 1 min and after 60 min incubation at ambient temperature (21°C ± 1°C), corrected for the absorbance decrease in a blank sample containing ABTS^•^ solution without any additive, was a measure of the antioxidant activity. The drop in absorbance after 1 min is assumed to be a measure of TAC conditioned by fast‐reacting antioxidants, the drop after 60 min as a measure of total TAC and the difference between these two values as a measure of TAC contributed by slow antioxidants (Kut et al. 2022).

### 2.5 Analysis of Polyphenolic Compounds Using UPLC‐PDA‐ESI‐MS/MS

Polyphenolic compounds were analyzed using a UPLC‐PDA‐ESI‐MS/MS Waters ACQUITY system (Waters, Milford, MA, USA) consisting of a binary pump manager, sample manager, column manager, photodiode array (PDA) detector, and tandem quadrupole (TQD) mass spectrometer with electrospray ionization (ESI), according to the method described (Kapusta et al. 2017). The separation was performed on a BEH C18 column (100 mm × 2.1 mm i.d., 1.7 m, Waters) maintained at 50°C. For the anthocyanins investigation, a solvent system of mobile phase A (2% formic acid in water, v/v) and mobile phase B (2% formic acid in 40% acetonitrile in water, v/v) was applied. For other phenolic compounds, a lower concentration of formic acid was used (0.1%, v/v). The gradient program was set as follows: 0 min, 5% B; from 0 to 8 min, linear to 100% B; and from 8 to 9.5 min, for washing and returning to initial conditions. The flow rate was 0.35 mL/min. The following parameters were used for TQD: capillary voltage 3.5 kV, cone voltage 30 V in positive and negative mode; the source was kept at 120°C, and the desolvation temperature was 350°C, con gas flow 100 L/h, and desolvation gas flow 800 L/h. Argon was used as the collision gas at a flow rate of 0.3 mL/min. Polyphenolic detection and identification were based on specific PDA spectra, mass‐to‐charge ratios, and fragment ions obtained after collision‐induced dissociation (CID). Quantification was achieved by the injection of solutions of known concentrations that ranged from 0.05 to 5 μg/mL (*R* ^2^ of 0.9998) of phenolic compounds as standards. All of the determinations were performed in duplicate and expressed as μg/mL. Waters MassLynx software v.4.1 (Waters, Milford, MA, USA) was used for data acquisition and processing.

### 2.6 Measurement of Fluorescence of Anthocyanin Oxidation Products

Rings of about 6 mm in diameter were cut from fresh and dried fruit, portions of ground dried fruit, and samples (40 mg) of the lyophilizate placed in wells of a 96‐well plate. Aliquots (200 μL) of the supernatants of ground fruit and homogenates were pipetted into wells of a plate. The top fluorescence measurement mode was employed. The slit width was 20 nm (both excitation and emission).

Fluorescence of all solid and fluid samples was measured in the range of 500–700 nm or 500–800 nm using an excitation wavelength of 460 nm in a Spark multimode microplate reader (Tecan Group Ltd., Männedorf, Switzerland).

### 2.7 Statistics

The results are presented as means±SD from measurements of at least three independent biological replicates for liquid samples and at least six biological replicates for solid samples, due to greater variability of results. The statistical significance of differences between antioxidants within individual assay methods was estimated using one‐way ANOVA with Tukey's post hoc test (*p* = 0.05) in XLSTAT, version 27.1.3.0 (Lumivero, Denver, CO, USA). PCA analysis was performed using STATISTICA, version 13.3 (TIBCO, Palo Alto, CA, USA).

## 3 Results

**FIGURE 2:**
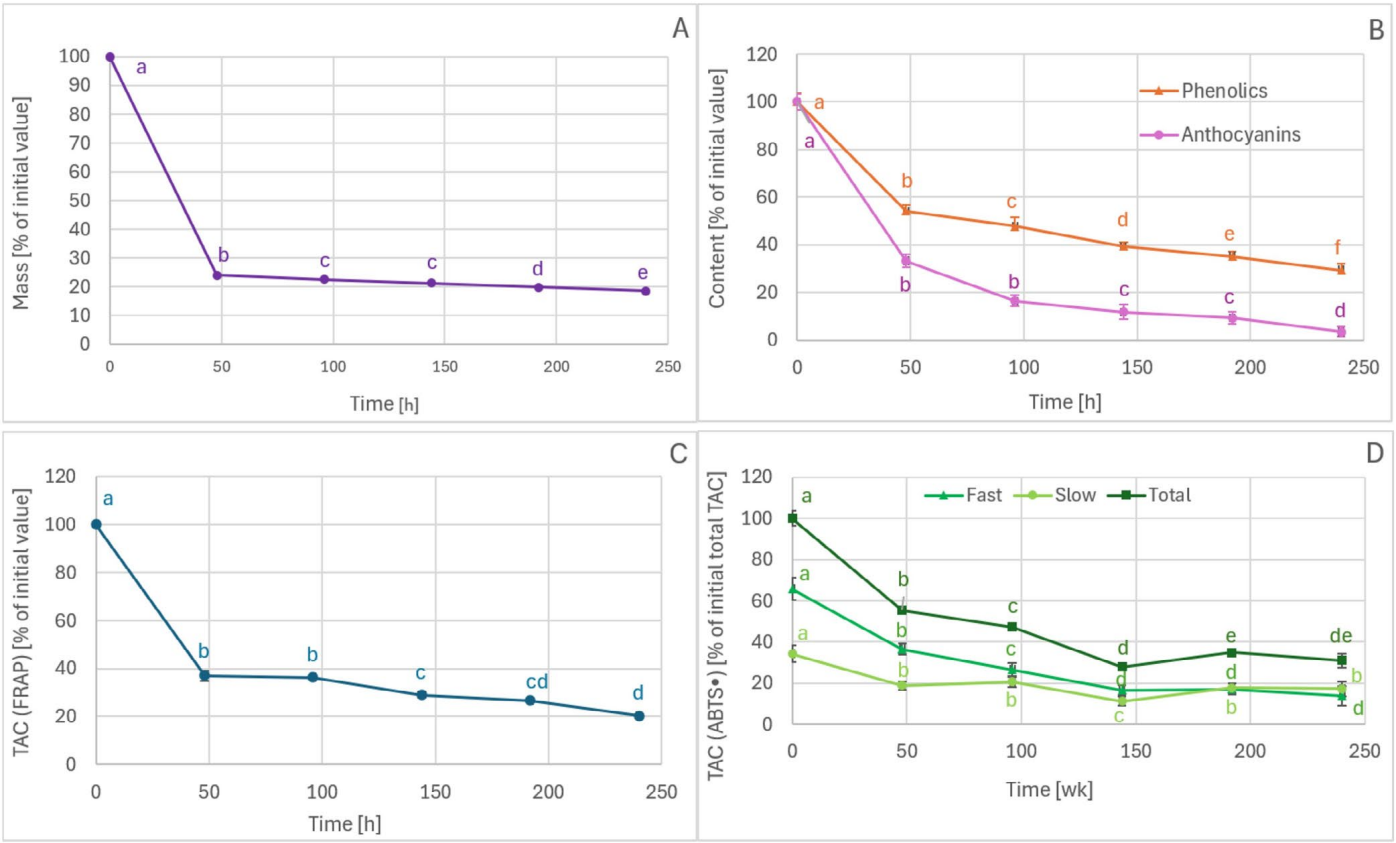
Decrease in sample mass (A) in the content of phenolics and anthocyanins (B) and of TAC estimated by FRAP (C) and ABTS^•^ decolorization (D) during the drying of aronia fruits. Initial values (assumed as 100% in each case): Phenolics, 15.7 ± 0.5 g GAE/kg initial mass; anthocyanins, 6.0 ± 0.2 g/kg initial mass; TAC (FRAP), 132 ± 2 mmol/kg; TAC (ABTS^•^), 191 ± 10 mmol/kg (fast), 99 ± 2 mmol/kg (slow), 290 ± 12 mmol/kg (total). In most cases SD smaller than the symbols. Different letter indices indicate a statistically significant difference (*p* < 0.05).

Heating the chokeberry fruit at 60°C was employed as a model situation of fruit drying. The mass of the fruit decreased considerably during 48 h, further drying leading to a further small mass decrease at a constant rate (Figure 2A). Prolonged heating resulted in a loss of phenolic and anthocyanin content, higher for anthocyanins than for total phenolics (Figure 2B). The loss of TAC estimated by FRAP (Figure 2C) and ABTS^•^ decolorization (Figure 2D) was similar to the loss of total phenolics, the fast reactivity (within the first minute of assay) being more pronounced than that of slow reactivity (Figure 2D).

The composition of phenolics analyzed by UPLC‐PDA‐ESI‐MS/MS is shown in Table 1. Four cyanidin derivatives, six quercetin derivatives, and three phenolic acids (chlorogenic acid, neochlorogenic acid, and cryptochlorogenic acid) were detected, anthocyanins being the dominant components.

**TABLE 1:** Individual phenolic compounds identified by UPLC‐PDA‐MS/MS in the ethanol chokeberry extract and their content in the fruits (mg/kg).

Compound		*Rt* (min)	*λ*_max_ (nm)	[M − H] *m*/*z*		Content (mg/kg)
				MS	MS/MS	
Anthocyanins						
1.	Cyanidin 3‐*O*‐glucoside	2.72	279, 515	449^+^	287	6117.26
2.	Cyanidin 3‐*O*‐galactoside	2.94	279, 514	449^+^	287	259.42
3.	Cyanidin 3‐*O*‐arabinoside	3.06	279, 515	419^+^	287	2648.66
4.	Cyanidin 3‐*O*‐xyloside	3.47	279, 515	419^+^	287	365.91
Other phenolics						
5.	Neochlorogenic acid	2.39	288sh, 324	353^−^	191	443.18
6.	Chlorogenic acid	3.00	288sh, 324	353^−^	191	465.31
7.	Cryptochlorogenic acid	3.34	288sh, 324	353^−^	191	103.60
8.	Quercetin 3‐*O*‐sophoroside	3.88	255, 354	625^−^	301	18.07
9.	Quercetin 3‐*O*‐glucoside‐pentoside	4.28	255, 354	595^−^	301	35.76
10.	Quercetin 3‐*O*‐rutinoside	4.51	255, 355	609^−^	301	26.90
11.	Quercetin 3‐*O*‐galactoside‐rhamnoside	4.60	255, 355	609^−^	301	26.35
12.	Quercetin 3‐*O*‐glucoside	4.69	255, 355	463^−^	301	71.91
13.	Quercetin 3‐*O*‐galactoside	4.81	255, 355	463^−^	301	44.26

**FIGURE 3:**
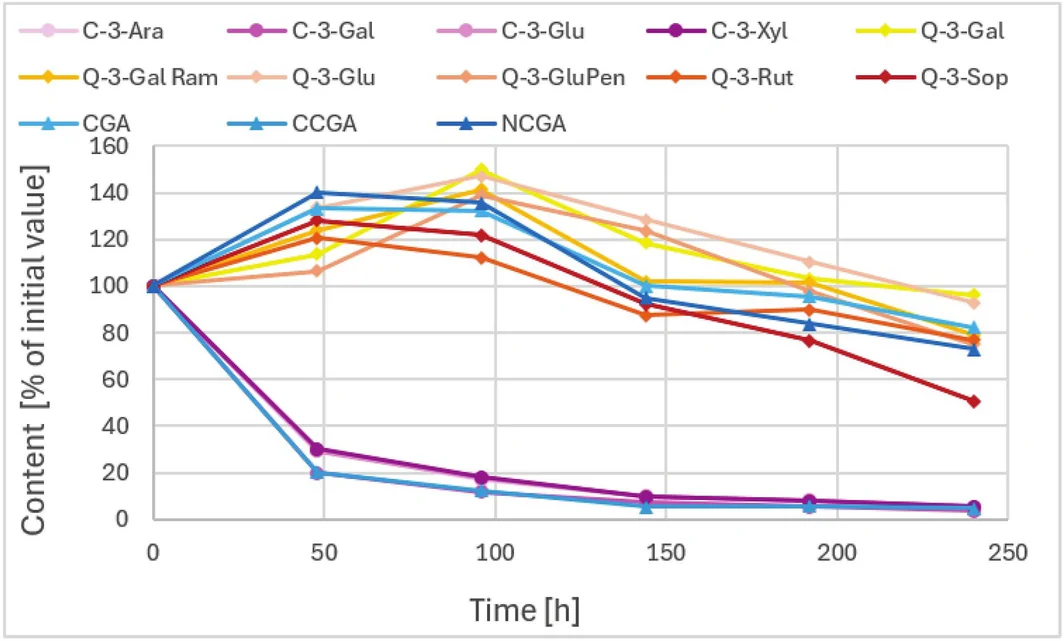
Changes in the content of individual phenolics during the drying of aronia fruits.

Changes in the concentrations of individual phenolics in the extracts of dried chokeberry fruits are shown in Figure 3. The decrease in the content of all anthocyanins was more pronounced than that of quercetin derivatives and phenolic acids, confirming and explaining the results shown in Figure 2A. It is also evident that the decrease was similar for more abundant and less abundant compounds within the individual classes of phenolics.

**FIGURE 4:**
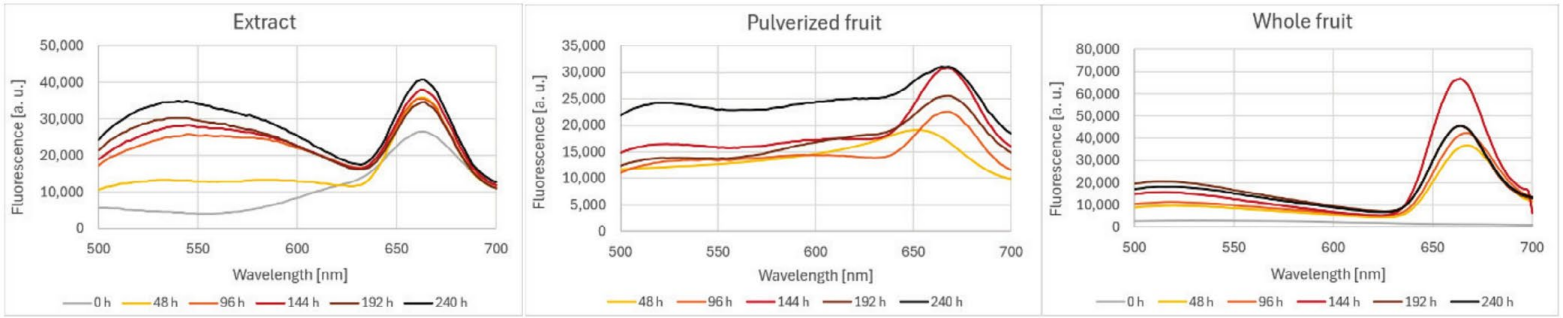
Fluorescence spectra of dried whole fruit, pulverized fruit and extracts of fruit dried for various times. Excitation wavelength: 460 nm.

Prolonged drying of chokeberry fruit led to the appearance and progressive growth of the fluorescence band characteristic of anthocyanin oxidation in the extracts, centered at 520–530 nm for the excitation wavelength of 460 nm. This band increased progressively with the heating time. Measurements of fluorescence of solid samples: whole dried fruit and pulverized fruit in wells of a 96‐well plate in a plate reader showed a less regular increase in the averaged spectra (Figure 4) and much higher variability of results.

**FIGURE 5:**
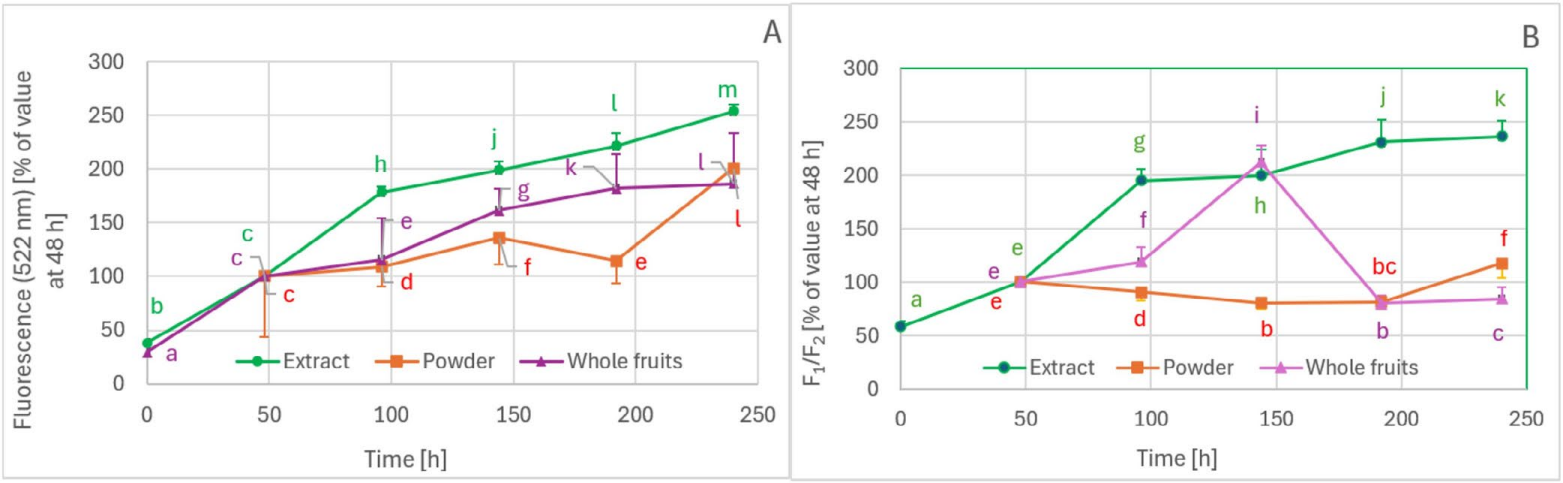
Effect of the drying time on the intensity of fluorescence characteristic for anthocyanin oxidation (*λ*_ex_, 460 nm; *λ*_em_, 522 nm) (A) and of the fluorescence intensity ratio at maximum 1 (about 522 nm) and maximum 2 (at about 530 nm) (B); Excitation wavelength, 460 nm. *p < 0.05, **p < 0.01, ***p < 0.001 with respect to the values at 1 week. Different letter indices indicate a statistically significant difference (*p* < 0.05).

A progressive increase in the maximal fluorescence intensity was observed for dried fruits, pulverized fruits, and fruit extracts during prolonged drying and was most regular for the fruit extracts (Figure 5A). The ratio of maximal fluorescence at the “oxidation band” (about 520–530 nm) to the maximal fluorescence at about 670 nm, typical of native anthocyanins (*F*_1_/*F*_2_ ratio), increased regularly in the extracts of fruits heated for prolonged time but not in solid samples of the chokeberry fruit and in pulverized fruit, where there was no increase in the *F*_1_/*F*_2_ ratio between 48 and 240 h of heating (Figure 5B).

**FIGURE 6:**
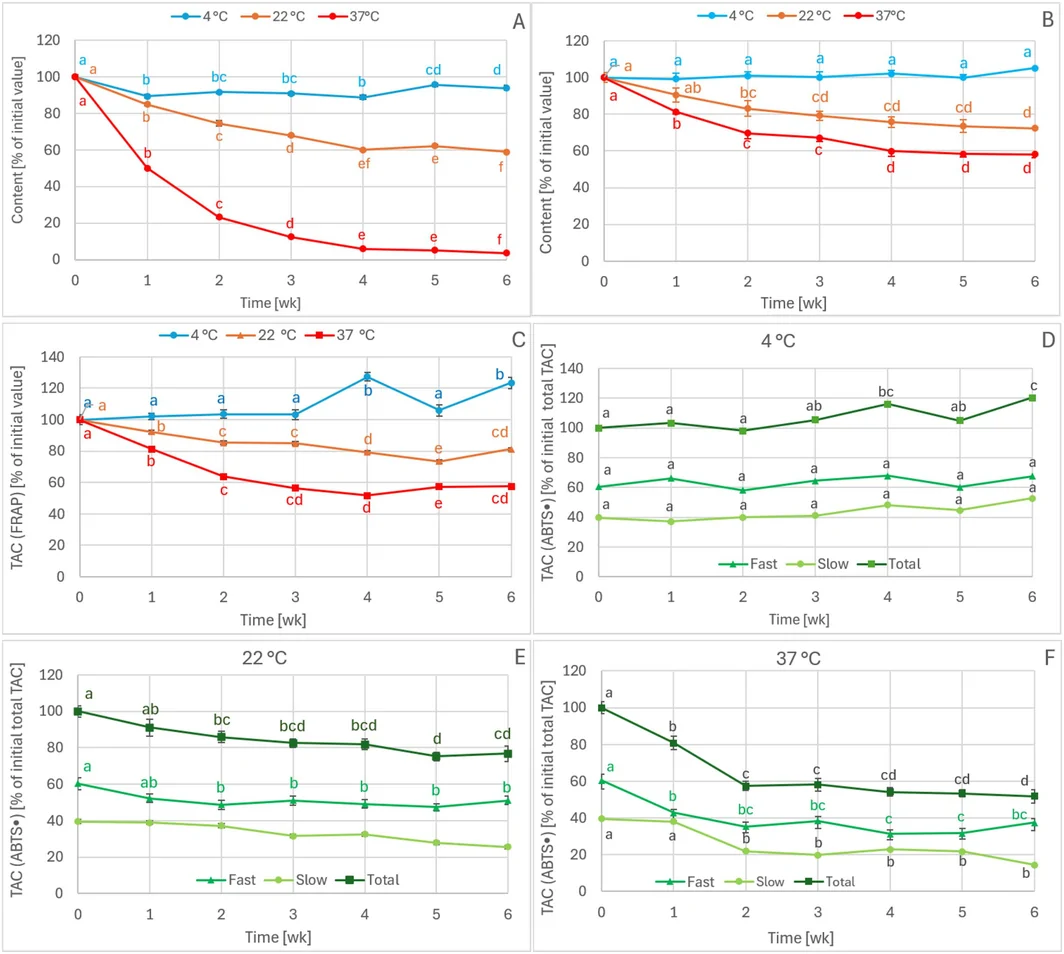
Effect of storage of lyophilizates of aronia fruits at various temperatures on the content of anthocyanins (A), total phenolics (B), and TAC estimated by FRAP (C) and ABTS^•^ decolorization (D–F). Initial values (assumed as 100% in each case): phenolics, 52.4 ± 1.9 g GAE/kg initial mass; anthocyanins, 18.8 ± 1.8 g/kg initial mass; TAC (FRAP), 1884 ± 23 mmol/kg; TAC (ABTS^•^), 461 ± 25 mmol/kg (fast), 302 ± 35 mmol/kg (slow), 763 ± 24 mmol/kg (total). Different letter indices indicate a statistically significant difference (*p* < 0.05).

Lyophilizates of chokeberry fruit stored in the presence of air showed no consistent decrease in the content of anthocyanins in lyophilizates when stored at 4°C, a significant progressive decrease during storage at 22°C and a profound progressive decrease during storage at 37°C (Figure 6A). The decline in the total phenolic content was similar but lower in magnitude (Figure 6B). Storage at 4°C did not decrease TAC estimated by both assays; storage at 22°C evoked a small decrease in TAC estimated by both assays, the decrease being contributed by fast‐reacting antioxidants; storage at 37°C caused a more pronounced decrease in TAC measured by both FRAP and ABTS^•^ decolorization.

The contents of individual phenolics in the lyophilizate extracts at “zero” time are shown in Table 2.

**TABLE 2:** Individual phenolic compounds identified by UPLC‐PDA‐MS/MS in the aqueous extract of lyophilizates of chokeberry fruits (mg/kg).

Compound		Content (mg/kg)
Anthocyanins		
1.	Cyanidin 3‐*O*‐glucoside	12,120.09
2.	Cyanidin 3‐*O*‐galactoside	339.35
3.	Cyanidin 3‐*O*‐arabinoside	4394.56
4.	Cyanidin 3‐*O*‐xyloside	580.20
Other phenolics		
5.	Neochlorogenic acid	1732.81
6.	Chlorogenic acid	1771.57
7.	Cryptochlorogenic acid	129.05
8.	Quercetin 3‐*O*‐sophoroside	30.26
9.	Quercetin 3‐*O*‐glucoside‐pentoside	88.40
10.	Quercetin 3‐*O*‐rutinoside	64.77
11.	Quercetin 3‐*O*‐galactoside‐rhamnoside	62.77
12.	Quercetin 3‐*O*‐glucoside	122.89
13.	Quercetin 3‐*O*‐galactoside	86.58

**FIGURE 7:**
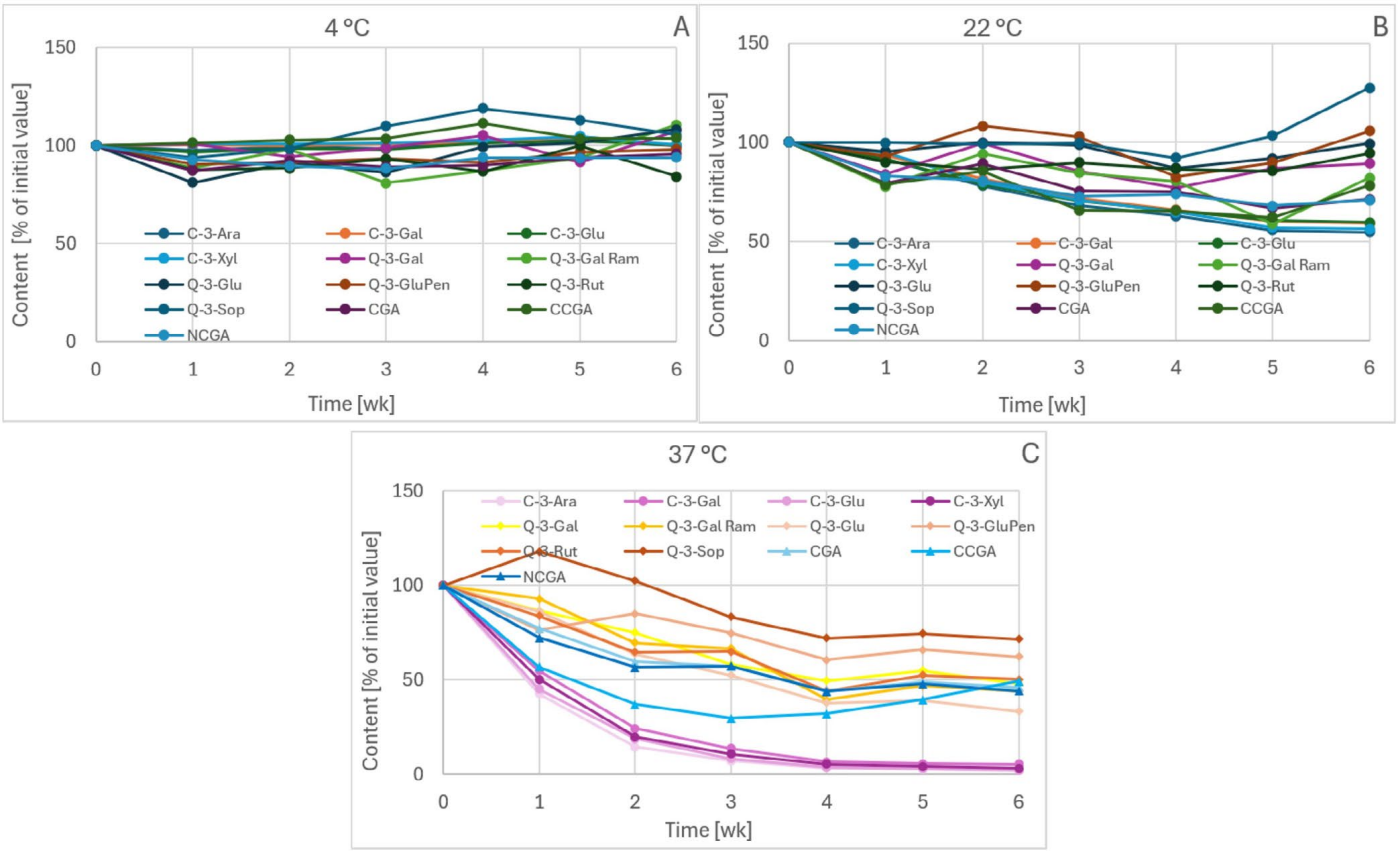
Effect of storage of lyophilizates of chokeberry fruits at various temperatures on the content of individual phenolics: 4°C (A), 22°C (B), 37°C (C).

Storage of the lyophilizate under conditions of air excess at 4°C did not change significantly the contents of individual phenolics (Figure 7A); storage at 22°C resulted in a small progressive loss of anthocyanins (Figure 7B), while storage at 37°C caused a distinct loss of anthocyanins and a smaller decrease in the content of other phenolics, similar for all anthocyanins irrespective of their initial content (Figure 7C).

**FIGURE 8:**
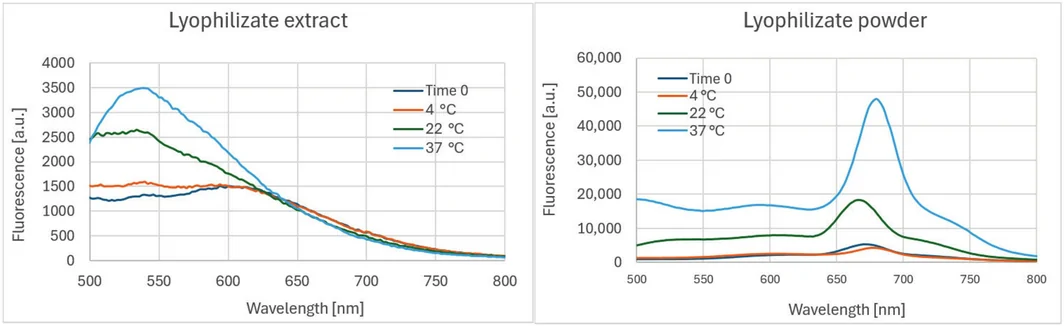
Fluorescence spectra of the lyophilizate extract and powder at time 0 and after various storage times at different temperatures for up to 6 weeks. Excitation wavelength: 460 nm.

The fluorescence spectra of the lyophilizate and the aqueous extract of the lyophilizate for the excitation wavelength of 460 nm differed significantly: a peak in the range of 520–540 nm (the “oxidation band”) was evident in the spectra of the extracts (Figure 8A), while a peak in the range of 660–680 nm dominated the spectra of the lyophilizate powder, being accompanied by two lower peaks below 600 nm (Figure 8B).

**FIGURE 9:**
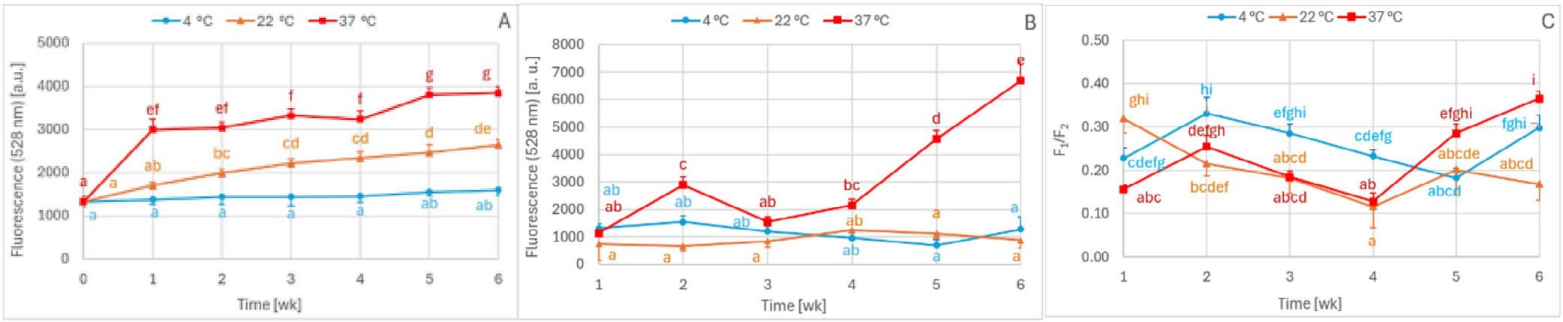
Changes in the intensity of fluorescence of lyophilizate extracts (A) and lyophilizate powder (B) at 528 nm, and the ratio of maximal fluorescence at peak 1 (about 528 nm) and peak 2 (about 670 nm) of the lyophilizate powder (C), during 6‐week storage at various temperatures. Excitation wavelength: 460 nm. Different letter indices indicate a statistically significant difference (*p* < 0.05).

The maximum fluorescence intensity at the anthocyanin “oxidation band” increased regularly in lyophilizate extracts, for lyophilizate stored at 22°C, starting from 2 weeks and, more profoundly, for lyophilizate stored at 37°C, starting from 1 week. No significant changes were seen in the fluorescence of lyophilizate stored at 4°C (Figure 9A). In the lyophilizate powder, an increase in the intensity of the “oxidation band,” not fully regular, was observed only for the lyophilizate stored at 37°C (Figure 9B). No consistent changes were observed in the ratio of maximum fluorescence intensities at the “oxidation band” and native fluorescence bands *F*_1_/*F*_2_ in the lyophilizate powder during storage at any temperature (Figure 9C).

## 4 Discussion

Although drying of chokeberry fruits is often indispensable for their storage and subsequent use, especially in the food industry (Calín‐Sánchez et al. 2015; Cristea 2016), it inevitably leads to a decrease in the content of their biologically active constituents, including phenolics and, especially, anthocyanins. The same phenomenon may occur during storage under nonoptimal conditions of chokeberry fruits even in the lyophilized form. Both heat‐drying of the chokeberry fruits at 60°C and storage of fruit lyophilizates with air excess at 22°C and, especially, at 37°C were found in this study to decrease the contents of total phenolics and anthocyanins and the TAC estimated by two assays. The decrease of the anthocyanin content was more pronounced than that of total phenolics (Figure 1), confirming that anthocyanins belong to the most fragile phenolics (Enaru et al. 2021; Woodward et al. 2009). Interestingly, the loss of anthocyanins was independent of their content in the samples, indicating a loss of selectivity in their degradation. This low stability of anthocyanins may, however, be their advantage, as they may thus be a good candidate for a marker of deteriorative changes in food products. A simple, ready‐to‐measure parameter of the anthocyanin state would be valuable. Fluorescence may be a promising technique in this respect.

Anthocyanins are weak fluorophores. Their fluorescence quantum yield is quite low (4.1 × 10^−3^ for 3,5,7‐trihydroxy‐2‐(4‐hydroxy‐3,5‐dimethoxyphenyl)chromenylium‐1‐ylium (malvidin) 3,5‐diglucoside) (Figueiredo and Pina 1990), mainly due to the efficient excited‐state proton transfer to water (Pina et al. 1998). For this reason, the fluorescence of anthocyanidins has not been extensively studied. Nevertheless, measurements of anthocyanidin fluorescence found applications, e.g., for the estimation of monomeric and polymeric anthocyanins in wine (Agati et al. 2013; Sadowska‐Bartosz and Bartosz 2025).

We observed the appearance of a new fluorescence band of anthocyanins upon oxidation of anthocyanins and anthocyanidins. The increase in the intensity of the fluorescence “oxidation band” of anthocyanins occurs upon the action of various oxidants, such as hydrogen peroxide, 2,2′‐azobis(2‐methylpropionamidine) (AAPH), 5‐amino‐3‐(morpholin‐4‐yl)‐1,2,3‐oxadiazol‐3‐ium (SIN‐1), sodium hypochlorite, and Fe(III) on these compounds. The appearance of such a band was not observed upon oxidation of other flavonoids, so it seems to be a universal marker of anthocyanin oxidation (Bartosz et al. 2020). The nature of the product(s) responsible for this fluorescence band is under study. However, regardless of its nature, this fluorescence band appears to be a useful parameter for monitoring the oxidation processes in various food products (Bartosz et al. 2020; Rak et al. 2025).

The fluorescence “oxidation band” of anthocyanins appears to be a candidate for a new, simple index of the state of chokeberry fruits or other anthocyanin‐rich food products. Its usefulness would be even greater if whole fruits or other solid products could be measured; such measurements would be completely nondestructive for the samples analyzed.

With this goal in mind, in this study, we compared changes in the fluorescence spectra of extracts of heat‐dried chokeberry fruits, pulverized as well as whole fruits, and extracts of fruit lyophilizates as well as the lyophilizate powder. We measured the solid samples in wells of a 96‐well plate in a standard plate reader.

Derivation of the *F*_1_/*F*_2_ parameter is possible when two fluorescence peaks are present in the spectra. This parameter was obtained from spectra of extracts of dried chokeberry fruits, pulverized fruits, and fruit lyophilizate but not from spectra of lyophilizate extracts since the native fluorescence peak was absent from the latter spectra.

While a regular increase in the intensity of the fluorescence “oxidation band” was observed in the extracts of dried chokeberry fruits and extracts of chokeberry fruit lyophilizates stored at higher temperatures, the results obtained for solid samples were more disappointing. An increase in the fluorescence intensity at the “oxidation band” was observed during heat‐drying for whole fruits, pulverized fruits, and lyophilizate stored at 37°C, but was not a fully monotonic function of time of heat‐drying or storage for pulverized fruits and the lyophilizate powder, respectively. The (*F*_1_/*F*_2_) ratio increased progressively during the drying time in the chokeberry fruit extract; its increase with the drying time was irregular for whole fruits, nonmonotonous for pulverized fruits, and completely irregular for the lyophilizate powder as a function of the time of storage.

It should be taken into account that measurements of solid samples in a multiplate reader are subject to considerable interference by light scattering, dependent on the variable geometry and surface structure of the samples. The small diameter of the exciting beam contributes to the variability of results due to the surface nonhomogeneity of solid samples (especially powders). Perhaps a portable fluorimeter, similar to those used for measurements of chlorophyll fluorescence in leaves, would be more suitable for this purpose. Out‐of‐equipment fluorescence measurements with a spectrofluorometer through a double‐arm optical fiber bundle were found to be useful for the monitoring of olive development and ripening (Agati et al. 2005).

A strange result is the lack of increase in the extracts of the content of any phenolic that could be a product of anthocyanin degradation. We suspect that these products were not extracted from the dried and lyophilized chokeberry fruits. Nevertheless, the fluorescence at the “oxidation band” increased in the extracts. The possible explanation is that anthocyanin oxidation induces a small change in the chromatographic properties of anthocyanins, not detectable by the separation system applied. Another possibility is that the fluorescence is due to a strongly fluorescent anthocyanin oxidation product formed in minute amounts.

Comparison of the phenolic content, anthocyanin content, and TAC of chokeberry fruits and their lyophilizates showed striking differences in the values of these parameters between extracts of dried fruits and of their lyophilizates, all of them being much higher when estimated based on concentrations measured in the lyophilizate extracts. The use of different solvents in both cases does not seem to be the reason for these differences, as, generally, ethanol has been reported to be somewhat more effective in the extraction of anthocyanins than water (Lapornik et al. 2005; Oancea et al. 2012). Apparently, the lyophilizate is a much better material for extraction of phenolics than dried chokeberry fruits.

The present results confirm that significant oxidation and degradation of anthocyanins occur during heat‐drying of chokeberry fruits and the storage of chokeberry fruit lyophilizates exposed to air at 22°C and, especially, 37°C. The lack of stability of anthocyanins in the fruit lyophilizates may seem surprising, but it should be taken into account that exposure to air is associated with exposure to oxygen and water vapor, creating conditions for oxidation and degradation processes in the material that becomes not completely dry. Practical conclusions from these results consist of recommendations: (1) not to extend the drying time of chokeberry fruits over the necessary minimum and (2) to store fruit lyophilizates tightly closed, without exposure to air.

## 5 Conclusions

A regular time‐dependent increase in the intensity of the “oxidation band” fluorescence was observed in the extracts of dried chokeberry fruit during prolonged heating and extracts of fruit lyophilizates during prolonged storage, indicating that it could be a preliminary indicator of anthocyanin oxidation. Direct fluorescence measurements of dried whole fruit, pulverized dried fruit, and lyophilizates were less reliable as indices of the time of drying and anthocyanin oxidation extent. The ratio of maximal fluorescence intensities at the “oxidation band” and native fluorescence band of extracts of dried chokeberry fruits is a ratiometric parameter potentially useful for the estimation of freshness of chokeberry fruits based on analysis of fruit extracts.

## Author Contributions

**Małgorzata Rak:** investigation (lead), validation (supporting), writing – original draft (supporting). **Grzegorz Bartosz:** writing – original draft preparation (supporting), writing – review and editing (supporting). **Kacper Kut:** investigation and validation (supporting). **Ireneusz Kapusta:** investigation and validation (supporting). **Maria Goc:** investigation (supporting). **Izabela Sadowska‐Bartosz:** conceptualization, methodology, resources, writing – original draft preparation (lead), writing – review and editing (lead), supervision, project administration, funding acquisition.

## Funding

This research was funded by the project “Modification of anthocyanins/anthocyanidins as new markers of food oxidation” (2023/51/B/NZ9/02490) financed by the National Science Centre (NCN), Poland, within the “OPUS 26” program.

## Conflicts of Interest

The authors declare no conflicts of interest.

## Data Availability

The data that support the findings of this study are available from the corresponding author upon reasonable request.
